# Erythrocyte glycophorins as receptors for *Plasmodium* merozoites

**DOI:** 10.1186/s13071-019-3575-8

**Published:** 2019-06-24

**Authors:** Ewa Jaskiewicz, Marlena Jodłowska, Radosław Kaczmarek, Agata Zerka

**Affiliations:** 1 Laboratory of Glikobiology, Ludwik Hirszfeld Institute of Immunology and Experimental Therapy, Polish Academy of Sciences, Rudolfa Weigla 12, 53-114 Wroclaw, Poland; 2 Faculty of Biological Sciences, University of Zielona Góra, Szafrana 1, 65-516 Zielona Góra, Poland

**Keywords:** Erythrocyte glycophorins A, B, C, D, *Plasmodium* EBA merozoite ligands, Receptor-ligand interaction, Malaria resistance

## Abstract

Glycophorins are heavily glycosylated sialoglycoproteins of human and animal erythrocytes. In humans, there are four glycophorins: A, B, C and D. Glycophorins play an important role in the invasion of red blood cells (RBCs) by malaria parasites, which involves several ligands binding to RBC receptors. Four *Plasmodium falciparum* merozoite EBL ligands have been identified: erythrocyte-binding antigen-175 (EBA-175), erythrocyte-binding antigen-181 (EBA-181), erythrocyte-binding ligand-1 (EBL-1) and erythrocyte-binding antigen-140 (EBA-140). It is generally accepted that glycophorin A (GPA) is the receptor for *P. falciparum* EBA-175 ligand. It has been shown that α(2,3) sialic acid residues of GPA O-glycans form conformation-dependent clusters on GPA polypeptide chain which facilitate binding. *P. falciparum* can also invade erythrocytes using glycophorin B (GPB), which is structurally similar to GPA. It has been shown that *P. falciparum* EBL-1 ligand binds to GPB. Interestingly, a hybrid GPB-GPA molecule called Dantu is associated with a reduced risk of severe malaria and ameliorates malaria-related morbidity. Glycophorin C (GPC) is a receptor for *P. falciparum* EBA-140 ligand. Likewise, successful binding of EBA-140 depends on sialic acid residues of N- and O-linked oligosaccharides of GPC, which form a cluster or a conformational structure depending on the presence of peptide fragment encompassing amino acids (aa) 36–63. Evaluation of the homologous *P. reichenowi* EBA-140 unexpectedly revealed that the chimpanzee homolog of human glycophorin D (GPD) is probably the receptor for this ligand. In this review, we concentrate on the role of glycophorins as erythrocyte receptors for *Plasmodium* parasites. The presented data support the long-lasting idea of high evolutionary pressure exerted by *Plasmodium* on the human glycophorins, which emerge as important receptors for these parasites.

## Background

Glycophorins, major sialoglycoproteins of human and animal erythrocytes, are transmembrane type 1 glycoproteins with relatively low molecular weight (20–30 kDa), carrying sialylated O-glycans and/or N-glycans [1, 2]. Despite these similarities, glycophorins show interspecific as well as intraspecific structural differences [3–5]. Human erythrocytes carry four glycophorins identified so far: GPA, GPB, GPC and GPD (Table 1). The structure and genetics of human glycophorins, as well as their common and rare genetic variants, are well known and have been the subject of several reports [6–19].

**Table 1:** Biochemical properties of human erythrocyte glycophorins [1, 2]

Glycophorin	No. of copies per cell	Length (aa)/MW (Da)	Number of	
			O-glycans	N-glycans
A	1 × 10^6^	131/43,000	16	1
B	2 × 10^5^	71/22,000	11	0
C	1.3 × 10^5^	128/39,000	12	1
D	5 × 10^4^	107/25,000	6	0

Glycophorins carry blood group antigens, including MN, Ss and Gerbich [1, 2]. Most GPA and GPB O-glycans are NeuAcα2-3Galβ1-3(NeuAcα2-6)GaINAc- tetrasaccharide chains linked to serine or threonine residues [15, 16], but truncated O-glycans (e.g. monosialylated linear trisaccharide, sialylated or nonsubstituted GalNAc residues) are also present. It is generally assumed that all human glycophorins contain similar O-glycans, while GPA and GPC also contain N-glycosidic chains at Asn26 and Asn8 residues, respectively. The structures of both N-glycans have been determined [17–19] and they contain complex biantennary chains with a bisecting GlcNAc and terminal sialic acid residues. In addition, GPC N-glycan contains small amounts of terminal fucose [19].

In recent years considerable progress has been made in the field of glycophorins as receptors for *Plasmodium* parasites. In particular, genome-wide association studies have shown that resistance to malaria may be connected with human glycophorin *ABE* locus [20], suggesting that glycophorins play an important role in erythrocyte invasion by *Plasmodium* malaria parasites.

Invasion of erythrocytes by *Plasmodium* parasites is a multistep process involving several ligands which enable the merozoite to gain entry to red blood cells (RBCs) [21–24]. Proteins belonging to two families, erythrocyte binding-like (EBL) and reticulocyte binding-like (RBL), have been identified as major determinants of erythrocyte invasion [25, 26]. Four functional EBL proteins have been found in *P. falciparum* merozoites, so far: erythrocyte-binding antigen-175 (EBA-175); erythrocyte-binding antigen-181 (EBA-181); erythrocyte-binding ligand-1 (EBL-1); and erythrocyte-binding antigen-140 (EBA-140) [27, 28]. All EBA ligands are transmembrane proteins and consist of six regions (I-VI) in their ectodomains. Two of these regions, II and VI, contain several conserved cysteine residues. Region II (RII) of *P. falciparum* EBA proteins comprises two homologous DBL domains in tandem: F1 (aa 8–282) and F2 (aa 297–603); in contrast, RII of *P.vivax* erythrocyte binding proteins contain only one DBL domain.

*Plasmodium falciparum* EBA-175 ligand [29–32] was the first and is the best characterized protein of the EBL family, considered to be one of the most important *P. falciparum* merozoite invasion ligands [32, 33]. It has been shown that EBA-175 is a target of human inhibitory antibodies present in sera of malarial patients, while animal antibodies recognizing EBA-175 can block merozoite invasion of RBCs [34–36]. Region II (616 aa) Pf EBA-175 was initially shown to mediate erythrocyte binding [31]. When truncated Regions I-VI of the Pf EBA-175 ectodomain were expressed on the surface of COS7 cells, only RII was bound by human erythrocytes in the rosetting assay. Furthermore, when domains F1 and F2 were expressed separately in COS cells, only F2 was shown to bind erythrocytes. The binding of F2 was similar to that of the entire Region II and to that of the full-length EBA-175, proving that the F2 domain of RII alone can facilitate erythrocyte binding. Because RII is highly conserved among laboratory and field isolates, it is considered as a potential vaccine candidate [26].

Evaluation of high-priority antigens and their receptors may serve as a means to rational design of novel therapeutics, which can inhibit binding of merozoites to erythrocytes during the blood stage of malaria [26, 37, 38]. However, the redundancy of EBA and RBL ligands, which enable the merozoite to use alternative RBC receptors and thus alternative invasion pathways, is one of the major obstacles to block invasion in a strain-transcending manner [24–26]. In this review, we concentrate on the role of glycophorins as erythrocyte receptors for *Plasmodium* parasites in general, and EBL merozoite proteins in particular.

### Glycophorin A as the receptor for *Plasmodium falciparum* ligands

It was shown that the EBA-175 ligand does not bind to erythrocytes treated with neuraminidase, which cleaves α(2,3)-linked sialic acids from the glycophorin oligosaccharide chains. In addition, *P. falciparum* parasites are unable to invade erythrocytes that lack glycophorins A and B (M^k^ phenotype). These data suggested that an erythrocyte membrane sialoglycoprotein (glycophorin A) may be the receptor for *P. falciparum* EBA-175 [29].

Moreover, binding of Pf EBA-175 to human GPB-negative erythrocytes (S-s-U-u-) and lack of binding to En(a-) erythrocytes, which are GPA-negative, confirmed that GPA is the receptor for EBA-175 ligand [31]. To investigate the binding site on GPA, tryptic (MT1 1–31, 1–39) and chymotryptic (MCH1 1–64, MCH2 1–34, MCH3 35–64) glycopeptides of the extracellular domain of GPA were used in EBA-175 binding inhibition assay [31]. It was found that binding of recombinant EBA-175 was inhibited only by glycopeptide MCH1 (aa 1–64), which contains 16 O-linked sialotetrasaccharides. The tryptic peptides MT1, containing 11 of the 16 oligosaccharide chains, as well as chymotryptic peptides MCH2 and MCH3, did not reveal significant binding inhibition, suggesting that in addition to the sialic acid residues, the presence of polypeptide chain of GPA is necessary for binding. Moreover, it was shown that GPB, which shares the first 26 aa residues with GPA and has 11 identical oligosaccharide chains, did not block EBA-175 binding. These data provided evidence that aa 1–64 of GPA play an important role in interactions with the ligand. Moreover, treatment of erythrocytes with N-glycanase did not affect rosetting of erythrocytes, demonstrating that the N-linked oligosaccharide does not play a role in EBA-175 binding. In summary, it is now generally assumed that GPA is the receptor for *P. falciparum* EBA-175 ligand, and α(2,3)sialic acid residues of O-linked glycosaccharides form conformation-dependent clusters on the GPA polypeptide chain which facilitate binding. However, other studies that included flow cytometry and ELISA analyses provided evidence that the EBA-175 ligand may also bind to desialylated GPA by engaging a 21-aa fragment (aa 1076–1096) of Region III and IV [39]. Evaluation of 11 truncated peptides of EBA-175 revealed that the peptide comprising aa 1085–1096 contains the erythrocyte binding site and strongly inhibits parasite invasion. Thus, it may be hypothesised that this EBA-175 peptide takes part in the second step of erythrocyte binding, which is a two-step phenomenon. The first step of this process is the initial binding of RII fragment to sialic acids of GPA, which induces a conformational change in EBA-175, which in turn exposes peptide (aa 1076–96) to subsequent erythrocyte binding. The ability of EBA-175 antigen to bind unsialylated GPA was later confirmed by other studies [40, 41].

The crystal structure of the erythrocyte binding domain of Pf EBA-175 (Region II) was resolved at 2.3 Å resolution, giving insight into the molecular mechanisms underlying the GPA binding [42]. The RII domain of Pf EBA-175 was crystallised in two distinct crystal forms, either as a monomer or as a homodimer, where two RII molecules arranged in an antiparallel fashion interact with each other in a manner resembling a handshake.

The centre of the homodimer contains two channels; most of the residues forming the channel surface come from the two F2 domains of the dimer, confirming its dominant role in the binding site of the EBA-175 ligand. In order to propose a model for the binding, the authors co-crystallized Region II with a glycan (α-2,3-sialyllactose) that may be considered as an analogue of Neu5Ac(α2,3)-Gal (the glycan receptor required for binding). The dimer interface contains six glycan binding sites: four of them are located inside the channel, while the remaining two are exposed in a cavity on the top surface. All of the six glycans contact residues of both monomers of Region II, implicating that binding of the receptor depends on dimerization. Computer modelling suggested that the O-glycosidic sialotetrasaccharides of GPA would fit in the binding sites with good geometry. A mutational analysis of RII suggested that all of the six glycan binding sites are necessary for RII binding. All these data helped to create a binding model of EBA-175 to GPA in which the binding induces dimerization of RII by assembling around the dimeric GPA extracellular domain. Alternatively, the GPA monomers could be bound to the outer surface of the RII dimer providing glycans for binding cavities.

The molecular basis of GPA and EBA-175 interaction involving the glycan moiety [Neu5Ac(α2,3)-Gal] is of particular interest when novel malarial therapeutics are considered. Indeed, it was shown that the sugar moiety, 2,3-didehydro-2-deoxy-*N*-acetylneuraminic acid (DANA), a structural analog of *N*-acetylneuraminyl-*N*-acetate-lactosamine, is a good inhibitor of Pf EBA-175 - GPA interaction (88% of inhibition at 100 μM) [43]. Moderate inhibition was also observed for monomers or oligomers of *N*-acetylneuraminic acid (from 25 to 68% at 100 μM). Moreover, it was shown that DANA is able to significantly inhibit erythrocyte invasion by *P. falciparum*. Thus, such compounds may one day be used as part of drug cocktails to reduce disease severity [43, 44].

A successful attempt to prevent formation of EBA-175-GPA complex involved the employment of monoclonal antibodies recognizing epitopes at glycan binding sites of RII/EBA-175 [45, 46]. Quantitative binding analysis revealed that a recombinant ectodomain of EBA-175 obtained in HEK cells interacts with GPA in a Neu5Ac(2-3)Gal-dependent manner with K_D_ 0.26 μm whereas a recombinant RII fragment alone bound GPA with a lower affinity [47]. Moreover, binding of RII to GPA and Neu5Ac(2-3)Gal showed similar affinity, whereas the full-length ectodomain of EBA-175 bound GPA with 2-fold higher affinity than the glycan alone. These results support the hypothesis that the RII fragment interacts with the GPA glycan moieties, but other extracellular regions of EBA-175 ligand outside of RII domain also engage the polypeptide of GPA.

**Fig. 1:**
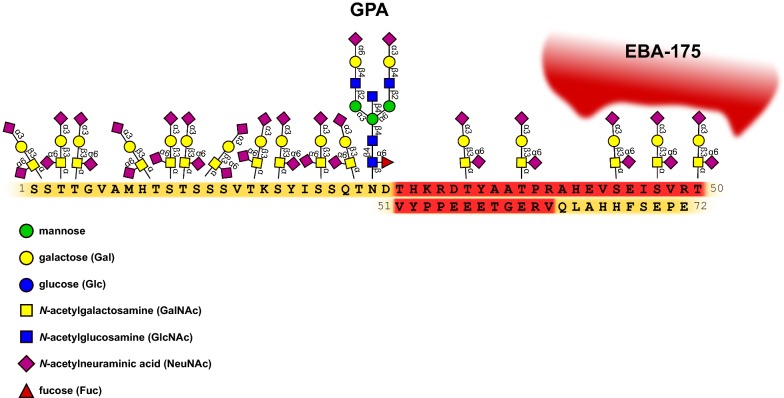
Amino acid sequence of external (aa 1–72) domain of GPA, its glycan structures and attachment sites [1, 4 modified]. GPA polypeptide fragment encoded by exon 3 is marked in red and its O-glycans recognized by EBA-175 ligand are shown

Recombinant GPA, produced in a mammalian expression system [48], was used to identify the GPA-RII binding region and evaluate the importance of its glycans for this interaction (Fig. 1). A recombinant GPA lacking the polypeptide fragment encoded by exon 3 did not bind EBA-175/RII. It was shown that presence of GPA multiple glycans is a prerequisite for high-avidity binding. Moreover, the glycosylation mutants had different effects on the binding. The triple glycan mutant, missing three glycans otherwise attached to Ser66, Ser69 and Thr72, did not bind to EBA-175 RII at all. On the other hand, the GPA individual mutants S69A and T72A revealed significantly reduced binding in comparison to S66A mutant, which bound with only minimally lower affinity than the wild-type GPA. Together, these results suggest that the three glycans of GPA polypeptide fragment encoded by exon 3 are critical for high-avidity binding of EBA-175 to GPA on erythrocytes. They are also consistent with the findings discussed earlier [31] demonstrating that digestion of purified, native GPA by trypsin (Arg39) within the sequence encoded by exon 3 abolishes its ability to inhibit erythrocyte rosette formation. In addition, it was shown before that M^g^ erythrocytes, in which GPA lacks glycans at aa residues 24, 25 and 26 [49], can be invaded by *P. falciparum* [50]. However, in other studies, a high frequency of variants with polymorphisms within exon 2 of the *GPA* gene was identified in three continental groups: sub-Saharan Africans, South Asians and Europeans. These results suggest that balancing selection has influenced exon 2, but not exon 3 of the *GPA* gene, in *P. falciparum* endemic regions [51].

Recently, new data emerged demonstrating an essential role of GPA-band 3 complex during the initial adhesion phase of malaria parasite invasion on RBCs [52]. It was shown that MSP1, which is highly abundant on the merozoite surface, binds to the trypsin-resistant (aa 31–72) extracellular domain of GPA. Moreover, deficiency of the Band 3-GPA-protein 4.2 complex in mouse RBCs was shown to be sufficient factor for resistance to invasion by *P. yoelli*. A similar effect was obtained for Band 3-GPA-band 4.2 null mouse RBCs infected with *P. falciparum* 3D7 strain *in vitro*. Based on these results, it was hypothesized that merozoites may use ligands that bind Band 3 (MSP1) and GPA (EBA 175) simultaneously, thus integrating distinct invasion pathways. Additionally, these findings suggested that MSP binding to GPA may change the RBCs membrane properties during parasite invasion [53].

These changes in the deformability of RBCs caused by EBA-175 binding to GPA were shown to activate phosphorylation of the cytoskeleton, which alters properties of the erythrocyte membrane and is required for successful invasion [54].

The most recent data concerning EBA-175-GPA interaction revealed its new role in promoting clustering of RBCs by EBA-175 antigen, shed post invasion, which is dependent on GPA [55]. This clustering not only provides merozoites ready access to uninfected RBCs but also protects from immune recognition. This phenomenon leads to enhanced merozoite growth and their immune evasion.

**Fig. 2:**
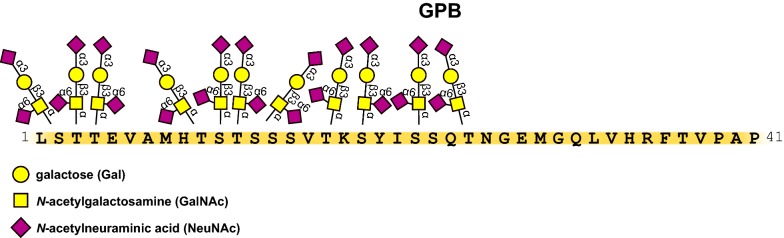
Amino acid sequence of external (aa 1–40) domain of GPB, its glycan structure and attachment sites [1, 4 modified]

In summary, the major invasion and evasion pathway of *P. falciparum* merozoites involves the interaction of EBA-175 and GPA. The observed patterns of variation in the *GPA* gene suggest that GPA has been subject to balancing selection in populations living in the malaria endemic areas of Africa and in Europeans [56]. However, when GPA is not accessible, *P. falciparum* can invade erythrocytes using different pathways, which may involve other glycophorins present on the surface of erythrocytes. One of them is GPB (Fig. 2), which is similar to GPA and has identical first 26 aa residues and O-glycosidic chains [1, 2].

### Glycophorin B as the receptor for *Plasmodium falciparum* EBL-1 ligand

Some isolates that depend on GPA for invasion can switch their invasion pathway in culture and invade GPA-deficient cells [57]. It was shown that *P. falciparum* can use an alternate pathway of invasion to enter human RBCs treated with trypsin, which digests GPA and GPC but not glycophorin B (GPB) (Fig. 2) [58]. Indeed, strains such as 3D7, HB3, Dd2 showed greatly reduced invasion rates for trypsin-digested S-s-U-RBCs (that lack GPB), indicating that the invasion depends on GPB [59]. However, the parasite ligand that binds to GPB remained unknown [60].

The early experiments showed that peptide fragments of EBL-1 merozoite ligand bound on human RBC membrane to a protein with an apparent molecular weight of 36 kDa [61]. It was also suggested that EBL-1 ligand can bind to GPB because its recombinant RII expressed on CHO-K1 cells did not bind to S-s-U RBCs, which lack GPB. In addition, the EBL-1 ligand immunoprecipitated from *P. falciparum* culture supernatant did not bind to S-s-U- RBCs. Moreover, EBL-1 ligand failed to bind to RBCs treated with chymotrypsin, which cleave GPB and neuraminidase, which removes sialic acid residues [62]. Thus, GPB emerged as a major candidate for EBL-1 binding. Statistical association between the presence of the S allele, encoding S antigen defined by presence of Met 29 residue in GPB [1, 10] and the infection rate suggested that GPB is a key receptor in the Brazilian Amazon regions [63]. This finding was supported by the unusually high occurrence of GPB-null phenotype in the Efe Pygmies in the Democratic Republic of Congo [64].

Similar to EBA-175 the RII region of EBL-1 ligand contains two DBL domains, F1 and F2. The major binding site of EBL-1 for GPB was identified within its 69-amino acid segment (F2i) of its F2 domain [65]. Both F1 and F2 domain and the F2i fragment of EBL-1 ligand partially inhibited the merozoite invasion of human RBCs. This result was confirmed by the finding that several peptides located within F1 and F2 domains of EBL-ligand bind to human RBCs [61]. Another proof that GPB is an important receptor for *P. falciparum* invasion came from screening of the merozoite and human RBC proteomes [66]. Of 13 predicted peptides, one peptide (SYTIRRLIKA) derived from human GPB showed the ability to inhibit parasite growth. These data may suggest that synthetic peptides may be considered to be included in the multi-subunit malaria vaccine [65].

Recently, using *ex vivo* cultured erythrocytes (cRBCs) with decreased expression of GPA, GPB or GPC, it was shown that most laboratory strains and field isolates of *P. falciparum* require GPB for invasion [67]. In the case of field isolates, GPB was found to be similarly important for invasion as GPA. Importantly, the observation that GPB is a key receptor in *P. falciparum* invasion is in contradiction to the fact that some laboratory strains have mutated or deleted the *ebl-1* gene [67].

### Glycophorin A-B hybrid (Dantu) as the receptor for *P. falciparum*

GPB-null phenotype is prevalent in the regions where malaria is endemic [68], suggesting that evolutional pressure exerted by *P. falciparum* caused selection for this polymorphism. Thus, the Dantu variant of GPB, which is a hybrid GPB-GPA molecule with a GPB N-terminus and GPA C-terminal region, seems to be responsible for protection against *P. falciparum* invasion *in vitro* [68, 69].

The large genetic association study (GWAS) of malaria, which included over 11,000 African children, revealed a new locus of resistance to severe malaria [70]. It was found close to the cluster of glycophorin genes *GPE*, *GPA* and *GPB.* Moreover, evaluation of genome sequence data from individuals of sub-Saharan descent suggested a strong protective effect of the Dantu (NE type) genotype against cerebral malaria anemia in East Africa [71]. The Dantu NE chromosome model contains *GypE*, *GypB* and *GypA* glycophorin genes; if translated, the encoded protein joins the extracellular domain of GPB to the transmembrane and intracellular domains of GPA, since *GPE* is probably not expressed at the protein level [72]. It creates the peptide sequence of Dantu NE blood group antigen, called DUP4. In addition to the Dantu variant, multiple deletions at the GPE-GPB-GPA locus were identified. The absence of exon 3 in cDNA of GPB and GPE limits specific recognition of GPA to EBA-175 merozoite ligand. One may even speculate that GPB and GPE arose due to selective pressure because the binding site on GPA has been lost.

The most recent studies of gene polymorphisms in children in Kenya confirmed that the presence of Dantu mutation was associated with reduced risk of severe malaria by 43% among heterozygotes and 74% among homozygotes, and the protection was equal for all forms of severe malaria. In addition, Dantu seemed to ameliorate malaria-related morbidity [73]. Moreover, studies in Tanzania indicated that DUP4 is susceptible to somatic variation and may be associated with increased haemoglobin levels [74]. These findings raised the question of how DUP4 protects against malaria. It may be hypothesized that it could affect interactions between *P. falciparum* receptors and host Band 3 at the RBC surface [73].

### Glycophorin C as the receptor for *Plasmodium* ligands

**Fig. 3:**
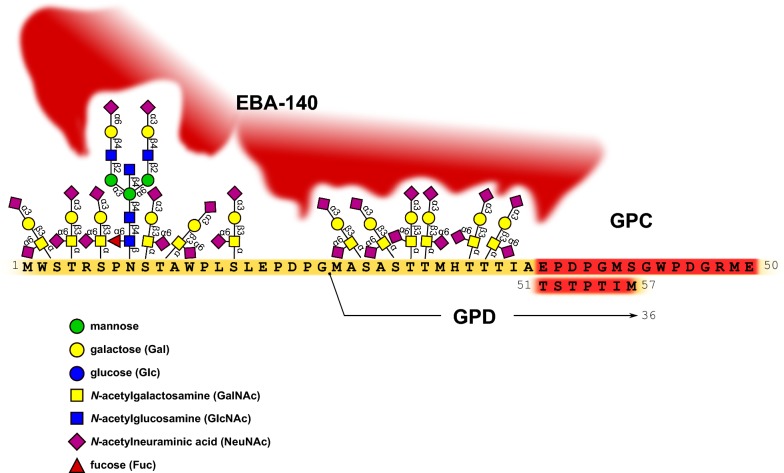
Amino acid sequence of external (aa 1–57) domain of GPC and GPD (aa 1–36) with their glycan structure and attachment sites [1, 4 modified]. GPC/GPD polypeptide fragment encoded by exon 3 is marked in red and putative glycans recognized by EBA-140 ligand are shown

Studies on the EBA-140 ligand, which similarly to EBA-175 contains two DBL domains F1 and F2 [27], showed that this ligand does not bind neuraminidase-treated human RBCs [75, 76]. However, RBCs lacking GPA [En(a-)], GPB (S-s-U-), or GPA and GPB [Dombrock, Gy(a-b-)] proved to bind EBA-140 effectively. Other RBC variants of the Yus and Gerbich phenotype, encoded by the genes lacking exons 2 or 3 in the *GYPC* gene [4] resulting in deletion of GPC amino acid residues 17–35 and 36–63, respectively, showed markedly reduced binding [75]. Moreover, only RBCs of the Leach phenotype (GPC null), lacking GPC, were not bound by EBA-140 ligand. Thus, these data suggested that the EBA-140 ligand binds human RBCs through GPC (Fig. 3). However, some contradictory results showed that (depending on the parasite strain) EBA-140 binding was not affected or only partially abolished when erythrocytes were treated with trypsin, which removes extracellular parts of glycophorins A and C [76, 77].

These inconsistencies may be attributed to the polymorphisms within the binding region of *P. falciparum* EBA-140 in different strains, which have been reported to alter host receptor specificity [78]. Five polymorphisms in the EBA-140 RII of different strains of *P. falciparum* have been identified (V,S,T,K; V,S,K,K; I,S,K,K; I,N,R,E; and I,N,K,K), which encompass four amino acid residues of the R II F1 domain in positions 185, 239, 261 and 285, respectively. These polymorphic variants of EBA-140 R II expressed on the surface of COS cells showed different receptor binding specificity. Of these five variants, only RII from the Dd2/Nm strain (V,S,T,K) was sensitive to neuraminidase and trypsin treatment of RBCs, and was the only one which did not bind to Gerbich-negative RBCs. A different binding pattern of two RII/EBA-140 polymorphic variants V,S,T,K and V,S,K,K was also confirmed in another study when both RII variants were expressed on the surface of Chinese hamster ovary (CHO-K1) cells [79]. It was demonstrated that only V,S,T,K (but not V,S,K,K) variant does not bind to neuraminidase treated RBCs or to Gerbich-negative erythrocytes. These data suggest that only one amino acid, T261 in V,S,T,K variant, may be the key residue for sialic acid-dependent GPC binding.

On the other hand, a study using native EBA-140 ligands from *P. falciparum* culture supernatants provided contradictory evidence [80]. All of the five polymorphic EBA-140 variants were proven to bind to GPC in a Western blotting assay with human erythrocyte membranes. None of these ligands bound to neuraminidase-treated or trypsin-treated RBCs or to Gerbich, Yus, Leach or Yus/Gerbich erythrocytes, which contain GPC variant forms or lack GPC completely. The authors came to the conclusion that the polymorphisms in EBA-140/R II does not change its specificity, but rather alter the affinity of binding to its receptor, which is GPC, and subsequently determine their ability to invade using this receptor. The discordant results obtained in the binding studies described above may be attributed to the use of different methodology. In contrast to the previous studies, which were based on recombinant forms of EBA-140 R II expressed on COS7 [78] or CHO-K1 [79], this study utilised native EBA-140 ligand from culture supernatants of different *P. falciparum* strains [80].

A decreased binding of EBA-140 observed in the case of Gerbich-negative erythrocytes [80–82] implied that the peptide encoded by exon 3 of *GPC* gene (aa 36–63) is of particular significance. However, these results were not confirmed in another study using EBA-140 from 3D7 clone supernatant (I,N,K,K), since this variant bound to Gerbich erythrocytes with a moderately higher efficiency than normal RBCs [83]. At the same time, the binding of EBA-140 to Yus cells was found to be decreased. It suggested that the amino acid residues 17–35, which Yus erythrocytes lack, but not 36–63, as previously suggested, play a significant role in invasion. However, what seemingly supports the hypothesis that aa 36–63 of GPC are of significance in merozoite invasion is the fact that frequency of the allele underlying Gerbich negativity has increased to almost 50% in populations where malaria is endemic, such as New Guinea [84]. Since *P. falciparum* parasites invade Ge-negative cells less efficiently than normal erythrocytes, it is highly possible that selection for this allele has been an evolutionary mechanism of protection against malaria. Determining which fragment of the polypeptide chain of GPC is responsible for binding EBA-140 ligand was the subject of several studies, also in our laboratory where we used RII/EBA-140 expressed in a baculovirus system. It confirmed that the GPC region encompassing aa residues 36–63 is indispensable for EBA-140 ligand binding [82].

Another issue to be solved is the role of the oligosaccharide chains of GPC in EBA-140 binding. The extracellular domain of GPC is believed to have 12 sialylated O-glycosidic chains linked to serine and threonine residues and one complex N-glycosidic chain at Asn8 [1, 2]. It was found that the N-linked glycan is crucial for EBA-140 binding of GPC [85]. It has been proposed that sialic acid residues of closely spaced N-linked and O-linked oligosaccharides of GPC form a cluster or a conformational structure that provides multivalency for the successful binding of EBA-140 (Fig. 3).

The turning point in the search for better understanding of the molecular basis of invasion by EBA-140 ligand came with solving the crystal structure of its recombinant binding region [86, 87]. It was shown that RII/EBA-140 is present as a monomer in solution in the asymmetric unit. The crystal structure of RII/EBA-140 bound to sialyllactose confirmed that this ligand-receptor complex exists as a monomer in solution, consistent with the crystal structures of unbound RII. Moreover, modelling revealed two receptor glycan-binding sites, each of them located in the F1 and F2 domain, in a structurally similar position. The two binding sites are present in a region that contains a high concentration of positively charged residues, presumably allowing the interaction with sialic acid which enters inside the binding pocket. It was suggested that domains F1 and F2 of R II/ EBA-140 act distinctly, and their role in receptor binding is different. It seems reasonable to assume that although both domains may be considered as required for binding, only domain F1 is involved in a direct interaction with the receptor.

In comparison to other RII regions of the DBL family, including homologous *P. falciparum* EBA-175 and *P. vivax* DBL ligand, the structure of PfEBA-140 RII and its glycan binding mechanism of PfEBA-140 is unique [87]. It was shown that DBL-domain receptor-engagement and glycan binding by PvDBP and PfEBA-175 ligands requires their dimerization [42, 88]. In addition, the PvDBP receptor binding site is formed through dimerization of the ligand upon receptor (DARC) binding [88, 89]. The formation of a homodimer is critical for and driven by receptor engagement. Consistently, RII-PfEBA-175 is known to exist as a monomer and dimerization is induced when binding with its receptor, GPA, occurs. Unlike the latter, RII of the PfEBA-140 ligand exists only in a monomeric form and consequently can bind only two GPC glycan moieties, whereas the PfEBA-175 RII dimer interacts with six O-linked glycans of GPA. These characteristics likely contribute to the increased affinity of the ligands to their receptors, GPC and GPA, respectively. Thus, whether *Plasmodium* ligands engage receptors as monomers or dimers is an issue of profound implications for parasite biology and control [42].

The structures of GPC N-glycans essential for GPC-EBA-140 binding were elucidated using mass spectrometry [90]. It was shown that apart from the complex-type sialylated structures, many GPC N-glycans contain terminal H2 blood antigen as well as polylactosamine chains with terminal fucose. It was hypothesized that presence of terminally fucosylated lactosamine units present in Gerbich-type GPC may explain the lack of EBA-140 binding to GPC Gerbich variant. Thus, it supported the theory about the crucial role of GPC sialylated N-glycan for GPC-EBA-140 interaction [85]. Moreover, it was shown that the *P. falciparum* EBA-140 ligand bound neither to Gerbich GPC deletion variant (lacking aa 36–63) nor to GPD (a truncated form of GPC lacking aa 1–21), but did bind to GPC Yus variant (lacking aa 17–35) [82]. It suggests that the heavily O-glycosylated N-terminal fragment of GPC (which also contains N-glycan) (Fig. 3) seems to be the binding site for EBA-140. It may be argued that deletion of the region including aa 36–63 in GPC Gerbich type, which in fact lacks O-glycans, might change the structure of GPC and influence the availability of other O-glycans and N-glycan attached to GPC. The essential role of sialylated oligosaccharide chains on GPC in EBA-140 binding is generally established, but the participation of individual O-glycans was not examined. In addition, the lack of EBA-140 binding to similarly glycosylated GPA (one N-glycan and 12–16 O-glycans) firmly suggests that GPC polypeptide also participates in the binding, either directly or by ensuring an aviability of glycans [82]. The crucial role of GPC N-terminus has been further corroborated by the prevalence of the rare Gerbich phenotype (Ge:-2,-3,4) in Papua New Guinea, which a *P. falciparum* endemic region [81, 84]. The molecular basis of these interactions may one day be a valuable means in the design of *P. falciparum* binding inhibitors for therapy.

In addition to the studies with the human *P. falciparum* parasite, interesting data recently came from studies using *P. berghei.* Because there is a significant homology in *GPC* genes across the species, the mouse GPC homolog was identified as a RBC receptor [91]. It was shown that the absence of GPC on mouse stem cell-derived RBCs resulted in a significant decrease of invasion rate by the mouse parasite (up to 66%) [91]. One homologous sequence to *P. falciparum* EBA-140 ligand in the *P. berghei* genome was identified. The putative protein called PBANKA-1332700 may serve as ligand for mouse GPC.

Once the parasite invades RBCs it releases variant surface antigens (var), such as erythrocyte membrane protein 1 (EMP1), STEVOR and RIFIN [92, 93]. Var-encoded proteins travel to the RBC plasma membrane and bind host proteins on uninfected RBCs and endothelium, which leads to sequestration of infected RBCs [93]. In addition, the rosettes form aggregates of uninfected and infected RBCs, which protect infected RBCs from the environment [94]. However, the importance of the rosetting occurrence *in vivo* remains unrecognized. It was revealed that GPC mediates rosetting by *P. falciparum* STEVOR protein [95]. Indeed, soluble GPC inhibits binding of STEVOR to RBCs simultaneously preventing rosetting. Moreover, GPC deletion significantly reduces the ability of RBCs to form rosettes.

Although most RBCs rosetting experiments were performed with *P. falciparum* infected RBCs, the general rule applies to *P. vivax* as well and it may also involve GPC. Surprisingly, rosettes formed by *P. vivax* occur preferably among normocytes (mature RBCs) and not reticulocytes [96], although *P. vivax* normally prefers to invade reticulocytes. In addition, a strong inhibition of *P. vivax* rosette formation by an antibody against the N-terminus of GPC was shown. Thus, GPC seems to function in the rosetting of *P. vivax.*

### Glycophorin D as the receptor on chimpanzee erythrocytes

As discussed above, there is a general agreement that GPC but not GPD is *P. falciparum* EBA-140 receptor on human RBCs [79, 81–83, 97, 98]. However, evaluation of the binding of the homologous *P. reichenowi* EBA-140 ligand binding to chimpanzee RBCs [99] unexpectedly demonstrated that the chimpanzee homolog of human GPD is probably the receptor for that ligand [4, 100]. It may be hypothesized that GPD is an ancestral EBA-140 receptor in non-human primates, while GPC emerged as a new receptor for *P. falciparum* in humans. This hypothesis needs to be corroborated using EBA-140 ligands from other species of *Plasmodium* invading Great Apes.

## Conclusions

Given the high death toll caused by malaria since time immemorial, it is not surprising that human genetic variants that protect against malaria are common in populations living in tropical areas. One example is the sickle cell allele that protects from malaria in heterozygous carriers, or Duffy antigen, which is a receptor for *P. vivax* that has been lost in many African ethnic groups rendering them resistant to *P. vivax* infection. *Plasmodium falciparum*, which causes most malaria cases in Africa, targets heavily glycosylated proteins that cover the surface of human RBCs, named glycophorins. Although GPA, GPB and GPC have been known as *P. falciparum* receptors for quite some time, data linking their polymorphism with resistance to malaria have been missing. With the identification of specific protective alleles in the glycophorin locus of individuals resistant to severe malaria, glycophorins have moved to the forefront of research on malaria epidemiology. Moreover, these data finally consolidate the long-standing idea that the human glycophorin makeup is a footprint of high evolutionary pressure exerted by *Plasmodium* parasites. Altogether, glycophorins emerge as not only an important foothold for the parasite on its way into the RBC but also an essential step in our quest to fully understand the *Plasmodium* biology and design new malaria interventions.

## Data Availability

All data presented and analyzed during this study are included in this published article.

## Abbreviations

GPA: glycophorin A
GPB: glycophorin B
GPC: glycophorin C
GPD: glycophorin D
aa: amino acids
